# The use of the HRM method for identifying possible mutations in the *ymoA* gene region and evaluating their influence on the enterotoxic properties of *Y. enterocolitica* strains

**DOI:** 10.1186/s12917-014-0207-6

**Published:** 2014-09-19

**Authors:** Agata Bancerz-Kisiel, Karolina Lipczyńska, Anna Szczerba-Turek, Eugenia Gospodarek, Aleksandra Platt-Samoraj, Wojciech Szweda

**Affiliations:** Department of Epizootiology, Faculty of Veterinary Medicine, University of Warmia and Mazury, Olsztyn, Poland; Department of Microbiology, Faculty of Pharmacy, Ludwik Rydygier Collegium Medicum in Bydgoszcz, Bydgoszcz, Poland

**Keywords:** *Yersinia enterocolitica*, *ymoA* gene, Yst enterotoxins, HRM, SNP

## Abstract

**Background:**

The *yst* gene that encodes the production of Yst enterotoxins is one of the most important and reliable virulence markers. Its ability to produce Yst has been demonstrated in pathogenic strains isolated from clinical cases of yersiniosis with diarrhea. However, not all *yst* positive strains produce enterotoxins. According to some authors, Yst production can be restored in a silent strain by *ymoA* mutation. In this study, the HRM method was applied to identify *ymoA* single nucleotide polymorphism with the aim of evaluating their influence on the enterotoxic properties of *Y. enterocolitica* strains.

**Results:**

Two genotypes (A and G) of the examined nucleotide sequence and some variations were detected in the HRM analysis. A phylogenetic analysis of 10 genotype A nucleotide sequences revealed 100% similarity with the *Yersinia enterocolitica* subsp. enterocolitica 8081 genome NCBI Acc. No. AM286415. An analysis of 10 genotype G nucleotide sequences and 3 variations sequences revealed two point mutations in the examined region: transition A3387326G and insertion A in position 3387368. However, no mutations were observed in the coding region of any of the examined *ymoA* gene fragments. Genotype G was identified in nearly all *Y. enterocolitica* strains isolated from pigs. Only 4 nucleotide sequences were similar to AM286415 and did not feature point mutations. In case of human *Y. enterocolitica* strains 31 were classified as belonging to genotype A, the remaining 59 belonged to genotype G and were characterized by the presence of point mutations.

**Conclusions:**

No correlations were observed between enterotoxic properties and the presence of mutations in the *ymoA* gene region of *Y. enterocolitica* strains isolated from both humans and pigs.

## Background

*Yersinia (Y.) enterocolitica* is an etiological agent of yersiniosis, a zoonotic disease that poses a growing threat for public health and produces a variety of clinical symptoms. However, not all *Y. enterocolitica* strains are pathogenic for humans and animals. Until recently, biotype and serotype diversity and the presence of pYV (plasmid Yersinia Virulence) were the key criteria for pathogenicity evaluation. Strains belonging to biotypes 1B and 2–5 *Y. enterocolitica* with pYV and chromosomal virulence markers were considered as pathogenic for humans and animals, but biotype 1A without classical virulence markers was regarded as non-pathogenic [1,2]. The most recent reports concerning clinical cases of yersiniosis in patients infected with *Y. enterocolitica* biotype 1A [3–5] indicate that the previous pathogenicity criteria should be revised.

The *yst* gene that encodes the production of Yst enterotoxins (*Yersinia* stable toxins) is one of the most important and reliable virulence markers. Its ability to produce Yst has been demonstrated in pathogenic strains isolated from clinical cases of yersiniosis with diarrhea. The mechanism of Yst action is based on guanylate cyclase activation, which results in increased cGMP levels in enterocytes and extracellular accumulation of liquid in the intestines [6]. However, not all *yst* positive strains produce enterotoxins. According to some authors, Yst production can be restored in a silent strain by *ymoA* mutation [7,8]. The *ymoA* gene encodes the YmoA (Yersinia modulator) protein, a member of the growing family of conservative Hha (hemolysin expression modulating) proteins with low molecular mass that are similar to H-NS (histone-like nucleoid structuring) group proteins [9]. H-NS proteins play an important role in intestinal microflora, both as structural proteins and gene expression modulators, including virulence markers [10]. The expression of genes directly responsible for the pathogenicity of *Y. enterocolitica* has been researched extensively. It is generally believed that YmoA is the key modulator of gene expression in response to environmental factors, including temperature [11,12]. The YmoA protein has also been shown to inhibit the expression of the *inv* gene encoding invasion, an important virulence factor responsible for the transport of bacterial cells across M cells [13] and participating in temperature-dependent production of *Yersinia* outer proteins (Yops) and *Yersinia* adhesin (YadA) – plasmid virulence markers [7]. The role of YmoA and related proteins (H-NS) in the regulation of gene expression in various microorganisms, including *E. coli*, has been established by numerous studies, whereas there are only few reports about *Y. enterocolitica* [7,9,11,13–15].

Single nucleotide polymorphism (SNP), referred to as the new generation genetic marker, is a DNA sequence polymorphism caused by single nucleotide (A, G, C, T) variation. The genotype distribution of the SNP site related to disease susceptibility can be analyzed to determine the correlation between a given genotype and susceptibility to disease, which provides a theoretical basis for disease prevention, personalized diagnosis and treatment [16]. Several SNP genotyping techniques have been developed, including polymerase chain reaction (PCR) followed by restriction fragment length polymorphism (RFLP), direct sequencing or PCR-based TaqMan chemistry. Some of those techniques are expensive, laborious and time-consuming because they support the analysis of only one SNP per reaction. High-resolution melting (HRM) analysis is a new and rapid method for the detection of mutations, in which PCR and mutation scanning are carried out simultaneously in a single procedure [17]. The detection of genetic variations in PCR amplicons requires HRM fluorescent dye and direct melting after PCR. The amplicon melting profile is determined by amplicon length, GC content, sequence characteristics and heterozygosity. This approach is a simple, closed-tube detection method that facilitates the identification of heteroduplexes and homoduplexes based on their characteristic melting curve profiles [18].

In this study, the HRM method was applied to identify *ymoA* SNP with the aim of evaluating their influence on the enterotoxic properties of *Y. enterocolitica* strains.

## Methods

An ethical approval was not required as this study was performed retrospectively. Samples of diseased humans were routinely submitted to the diagnostic laboratories (National Institute of Public Health – the National Institute of Hygiene in Poland and Department of Microbiology, Ludwik Rydygier Collegium Medicum in Bydgoszcz). The collection of *Y. enterocolitica* strains isolated from pigs was obtained from previous studies, performed in adherence to the Local Ethics Committee of the University of Warmia and Mazury in Olsztyn.

### Experimental material

The material for the study consisted of 90 *Y. enterocolitica* strains isolated from clinical cases of human yersiniosis accompanied by diarrhea (mostly from the collection of the National Institute of Public Health – the National Institute of Hygiene in Poland) and 90 *Y. enterocolitica* strains isolated from clinically healthy fattening pigs. Both human and pig strains of *Y. enterocolitica* had been previously biotyped, serotyped and molecularly examined (multiplex PCR – *ystA, ystB, ystC, ymoA*) [19]. Determination of enterotoxic properties *Y. enterocolitica* isolated from pigs was done using suckling mouse bioassay, described previously [19]. Enterotoxin production has been evaluated semi quantitatively by the measurement of the ratio of intestinal mass to the rest of the body mass in the group of three examined sucklings. According to Gianella [20] ratio ≤ 0.074 was regarded as a negative result, 0.075 – 0,082 as a doubtful result and ratio ≥ 0.083 was regarded as a positive result. In this study 14 positive, 33 doubtful and 43 negative *Y. enterocolitica* strains were used. All the *Y. enterocolitica* strains isolated from clinical cases of yersiniosis produced enterotoxin, what resulted in diarrhea.

### HRM analysis

The HRM analysis was performed in the Rotor-Gene 6000™ real-time analyzer (Corbett Life Science, Sydney, Australia) using the PCR HRM curve analysis assay. PCR HRM was conducted with the use of the Eva Green saturating dye (Type-it HRM PCR Kit, Qiagen, Hilden, Germany). Sequences of primers used in the reaction: *ymoA*-1 (Forward) 5′GACTTTTCTCAGGGGAATAC3′ and *ymoA*-2 (Reverse) 5′GCTCAACGTTGTGTGTCT3′ were previously published by Grant et al. [8]. PCR was performed in 25 μl reaction volumes containing 12.5 μl of 2× HRM PCR Master Mix, 10.15 μl of RNase-free water, 1.75 μl of the primer mix (final concentration 0.7 μM each) and 0.6 μl of DNA (50 ng/reaction). PCR amplification was performed with initial denaturation at 95°C for 5 min, followed by 40 cycles at 95°C for 10 s and 55°C for 30 s. HRM ramps were generated by acquiring fluorescence data at the temperature ramp of 65°C to 90°C at 0.1°C intervals. HRM curves were normalized, and the genotype was assigned based on the shape of the HRM curve with the use of the Rotor-Gene software and by visual examination.

### Data analysis

The observed melting curves were analyzed using RotorGene 6000 Series Software 1.7. Ten samples of each of the two SNP genotypes and 3 variations were randomly chosen for sequencing to verify genotyping results (Genomed Sp. z o.o., Warsaw, Poland). Sequence data from the examined *Y. enterocolitica* strains were compared with the nucleotide sequence of the previously identified *ymoA* gene, lodged in National Centre for Biotechnology Information (NCBI, position 3387372–3387575 in the *Y. enterocolitica* Acc. No. AM286415) using BLASTN version 2.2.18. [21]. Multiple sequence alignment was carried out in ClustalW [22] incorporated in the freeware Computational Evolutionary Biology package MEGA version 5.2.1. [23]. Nucleotide sequences were demonstrated using BioEdit v.7.2.0. software.

## Results

**Figure 1:**
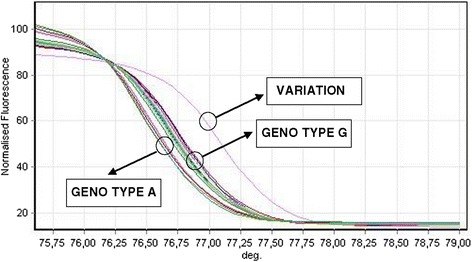
**HRM normalized curve of** ***Y. enterocolitica ymoA*** **gene.** Figure shows 6 genotypes A, 17 genotypes G and one variation of the examined nucleotide sequence *ymoA* gene *Y. enterocolitica* strains isolated from human cases of yersiniosis detected using HRM.

**Figure 2:**
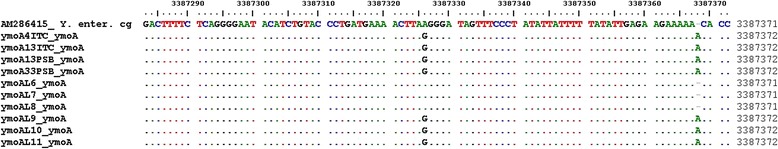
**Single nucleotide polymorphism of** ***Y. enterocolitica ymoA*** **gene** ***.*** A phylogenetic analysis of 3 genotype A nucleotide sequences *ymoA* gene *Y. enterocolitica* strains isolated from human cases of yersiniosis (L6, L7, L8) revealed 100% similarity with the *Y. enterocolitica* AM286415 sequence. An analysis of 7 genotype G nucleotide sequences *ymoA* gene (3 *Y. enterocolitica* strains isolated from human cases of yersiniosis – L9, L10, L11 and 4 isolated from pigs – 4ITC, 13ITC, 13PSB, 33PSB) revealed two point mutations in the examined region: transition A3387326G and insertion A in position 3387368.

Two genotypes (A and G) of the examined nucleotide sequence and some variations were detected in the HRM analysis (e.g. Figure 1). Direct sequencing revealed that examined nucleotide sequences had the length of 319 bp (base pairs) according to the NCBI. They were linked in position 3387283–3387600 in the *Yersinia enterocolitica* subsp. enterocolitica 8081 genome NCBI Acc. No. AM286415, where the gene responsible for the production of a conserved hypothetical protein (NCBI, Protein ID CAL13149) is encoded in position 3386958–3387326, and the *ymoA* gene responsible for the production of histone-like protein YmoA (NCBI, Protein ID CAL13150) is encoded in position 3387372–3387575. A phylogenetic analysis of 10 genotype A nucleotide sequences revealed 100% similarity with the *Y. enterocolitica* AM286415 sequence. An analysis of 10 genotype G nucleotide sequences and 3 variations sequences revealed two point mutations in the examined region: transition A3387326G and insertion A in position 3387368 (Figure 2). Transition A3387326G was related to the stop codon (TAA»TAG) of the gene encoding conserved hypothetical protein CAL13149. Insertion A was noted in the non-coding region of the investigated area, and it did not change the reading frame for the start codon of the *ymoA* gene in position 3387372. No mutations were observed in the coding region of any of the examined *ymoA* gene fragments.

Two SNPs (transition A3387326G and insertion A3387368) were identified in the examined sequence in nearly all *Y. enterocolitica* strains isolated from pigs. Only 4 (3.6%) nucleotide sequences were similar to AM286415 and did not feature point mutations. Genotype A strains belonged to bioserotype 4/O:3 – one of them was isolated from the toxin-producing *Y. enterocolitica* strain, two were isolated from doubtful strains, and one – from a strain that did not produce toxins in a study of suckling mice. No correlations were observed between enterotoxic properties and the presence of mutations in the examined sequence of *Y. enterocolitica* strains isolated from pigs. More than 27.0% of *Y. enterocolitica* strains from clinical cases of human yersiniosis were similar to nucleotide sequences of *ymoA* gene *Y. enterocolitica* AM286415. A total of 31 human *Y. enterocolitica* strains were classified as belonging to genotype A, including 14 strains belonging to bioserotype 1B/O:8 and 17 strains belonging to bioserotype 4/O:3. The remaining 59 human *Y. enterocolitica* strains of bioserotype 4/O:3 belonged to genotype G and were characterized by the presence of point mutations.

## Discussion

In the present study, potential mutations in the *ymoA* gene associated with the pathogenesis of yersiniosis were identified by HRM analysis. Genotype data from *Y. enterocolitica* strains with confirmed enterotoxic properties were compared to data from *Y. enterocolitica* strains that do not produce enterotoxins. Amplicon genotyping by HRM analysis supported rapid identification of the frequencies of *ymoA* SNPs suspected of influencing the *yst* gene function.

The *ymoA* mutation and its possible effects on the *yst* gene were first described by Cornelis et al. [7] who identified two Tn5-Tc1 chromosomal insertion mutants of W22703 transcribing classical virulence markers at low temperature. Those mutants also resumed their production of Yst, with its typical temperature dependence. Both mutations were insertions in the same gene called *ymoA* for ‘Yersinia modulator’. The cloned *ymoA* gene fully complemented the two mutations. The cited authors suggested that the *ymoA* mutation unblocks the silencing of the *yst* gene and stimulates enterotoxin production and that the *ymoA* gene without mutation is responsible for negative regulation of *ys*t gene expression.

In 1994, Mikulskis et al. [24] suggested the presence of a mechanism switching the expression of *yst* to a silent state. According to the above authors, gene silencing was triggered by changes in the status of bacterial host factors rather than modifications in the *yst* gene. They also noted that negative regulator YmoA participated in *yst* silencing and temperature regulation of *yst.* YmoA was regarded as necessary for growth-phase regulation of *yst*, although it was not the only factor involved in the regulation process.

In 1998, Grant et al. [8] also demonstrated that *yst* gene carriage is not always correlated with the presence of the toxin in culture supernatants, and similar results were noted in our previous studies [19]. Both cited studies suggested that the absence of enterotoxic properties could result from reduced gene expression in *in vitro* cultures, possibly by *ymoA*.

The cited results differs from observations made in this study. Instead of the two insertions reported by Cornelis et al. [7], this study revealed transition A3387326G in the stop codon of the gene fragment encoding protein CAL13149 and insertion A3387368 in the non-coding region of the *ymoA* gene. No mutations were observed in the coding region of any of the examined *ymoA* gene fragments.

Two SNPs detected in this study were observed in *Y. enterocolitica* strains, regardless of their enterotoxic properties, isolated from both humans and clinically healthy pigs. Genotype G characterized by those SNPs accounted for 96.4% of strains isolated from pigs and 72.1% of strains isolated from humans. Genotype A without point mutations was observed far less frequently, but was detected in 100% bioserotype 1B/O:8 strains isolated from humans, which are believed to be the most pathogenic causative agents of acute yersiniosis [25].

## Conclusions

The results of this study indicate that two point mutations in the analyzed nucleotide sequences do not affect the toxic properties of the examined strains. It should also be noted that the absence of mutations in the coding region of the *ymoA* gene does not confirm their influence on *ystA* gene silencing, which was postulated by other authors. The possible role of YmoA in the negative regulation of *yst* genes should be analyzed through simultaneous measurements of *yst* and *ymoA* gene expression levels.

## Abbreviations

*Y. enterocolitica*: *Yersinia enterocolitica*
pYV: Plasmid *Yersinia* Virulence
Yst: *Yersinia* stable toxins
cGMP: Cyclic guanosine monophosphate
YmoA: Yersinia modulator
Hha: Hemolysin expression modulating protein
H-NS: Histone-like nucleoid structuring protein
Inv: *Yersinia enterocolitica* invasin
Yops: *Yersinia* outer proteins
YadA: *Yersinia* adhesin
*E. coli*: *Escherichia coli*
SNP: Single nucleotide polymorphism
PCR: Polymerase chain reaction
RFLP: Restriction fragment length polymorphism
HRM: High-resolution melting
NCBI: National Centre for Biotechnology Information
bp: Base pair
